# Supplementary material for: Quantifying protein dynamics and stability in a living organism
Source: Nat Commun. 2019 Mar 12;10:1179. doi: 10.1038/s41467-019-09088-y (PMC6414637; doi:10.1038/s41467-019-09088-y)
Supplement: Supplementary file 1 — Supplementary Information [file 41467_2019_9088_MOESM1_ESM.pdf]

**<u>Supplementary Information for</u>**

# Quantifying protein dynamics and stability in a living organism

Ruopei Feng *et al.*

## Supplementary Figures

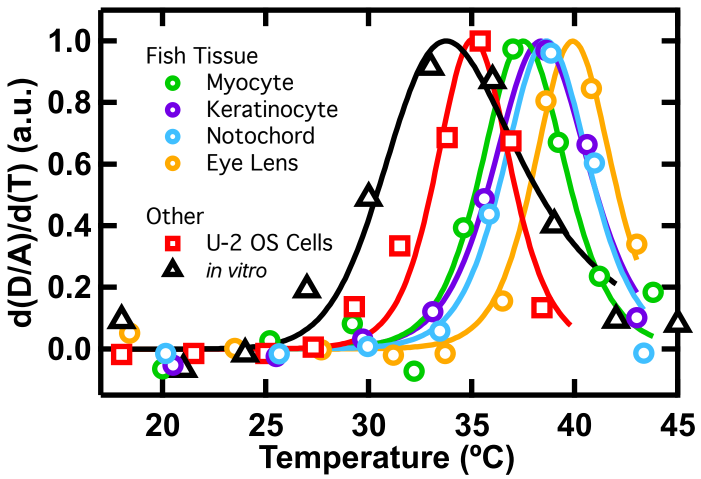


**Supplementary Figure 1**. Representative thermal denaturation of PGK3 89-4G FRET monitored by fluorescence microscopy in zebrafish tissues (circles): myocyte (green), keratinocytes (purple), notochord (blue), or eye lens cells (orange); U-2 OS mammalian cells (red squares); or in vitro (black triangles). The signal from two-color FRET experiments is reported as donor/acceptor ratio. The data in Fig 3A was algebraically differentiated to yield the differential melting curves. Solid lines indicate the best fits to Equations 3 to 5. Source data are provided as a Source Data file.

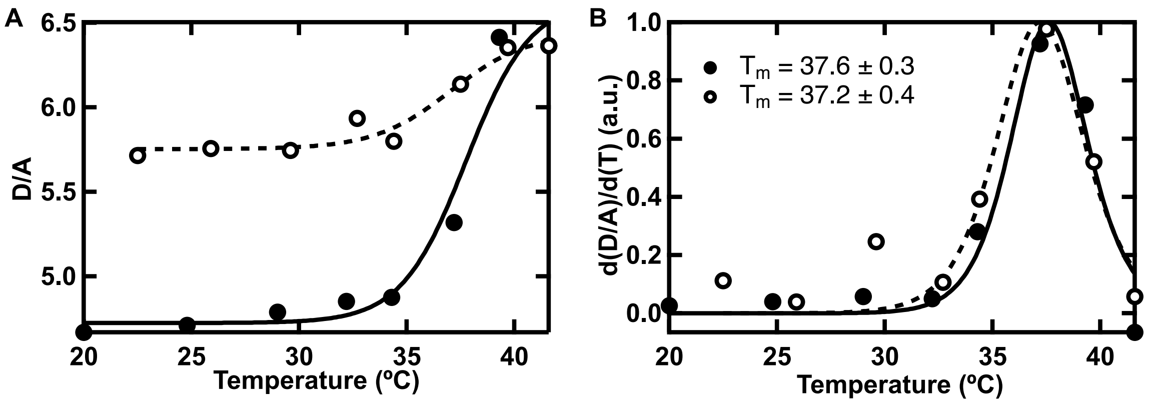


**Supplementary Figure 2.** Representative reversibility following a thermal denaturation of PGK3 89-4G FRET in myocytes of zebrafish. The signal from two-color FRET microscopy experiments is reported as donor/acceptor ratio. **(A)** Complete denaturation (solid circles) and refolding (open circles) profiles between 18-45 °C. A reversibility of 38% is calculated by the ratio of the difference in the maximum and minimum baselines of the refolding and denaturation curves ($[Max_{refolding}$-$Min_{refolding}]$/ $[Max_{denatured}$-$Min_{denatured}]$). A sigmoidal fit is overlaid on the data. **(B)** The data in (A) were algebraically differentiated to yield the differential melting curves. Solid lines indicate the best fits to equations 3 to 5. Thermodynamic parameters derived from the fits are within error, supporting our assessment of reversibility. Data are presented as mean ± SD. Source data are provided as a Source Data file.

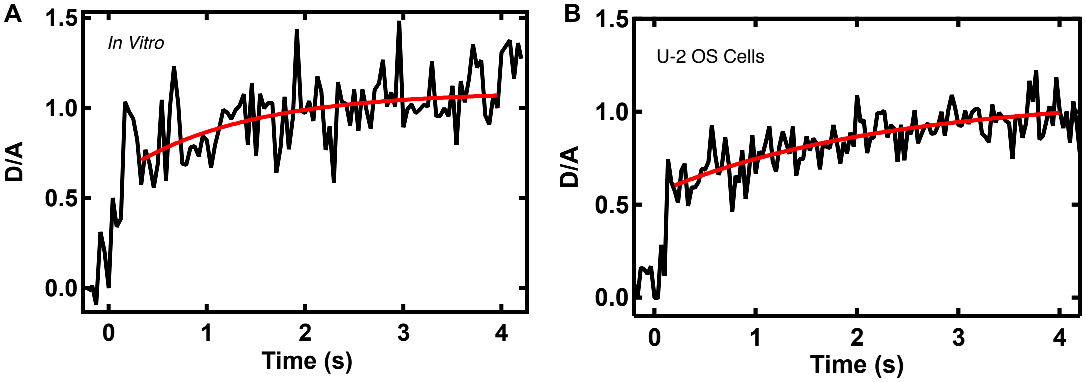


**Supplementary Figure 3.** Representative relaxation kinetics of PGK **(A)** *in vitro* and in **(B)** U-2 OS cells following a jump to $T_m$ obtained by fluorescence microscopy. The signal from two-color FRET experiments is reported as a donor/acceptor ratio. A single exponential fit (Equation 6) is overlaid on the data between 0.2 and 4 s. Source data are provided as a Source Data file.

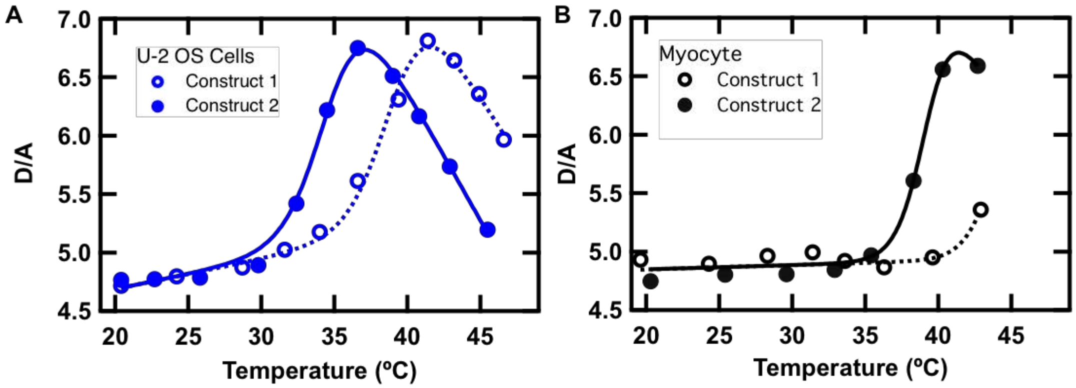


**Supplementary Figure 4.** Representative thermal denaturation of FRET-PGK with (Construct 2, shown as solid circles) and without (Construct 1, shown as open circles) the loop mutation monitored by fluorescence microscopy. The signal from two-color FRET experiments is reported as donor/acceptor ratio. The denaturation profile between 18-45 °C monitored in U-2 OS cells **(A)** and zebrafish myocyte **(B)**. Each panel is globally fit to a sigmoid with a different $T_m$, the same $\Delta H$, and the same pre- and post- transition baselines. Note that in (B) only the onset of unfolding is observed for construct 1. Source data are provided as a Source Data file.

## Supplementary Tables

**Supplementary Table 1.** Thermodynamic parameters and standard deviations derived from a two-state fit of the equilibrium myocyte data.

| Myocyte Cell # | Organism # | $T_m$ (°C) | $\Delta H$ (kcal mol$^{-1}$) |
|---|---|---|---|
| 1 | Fish 1 | 37.0 ± 0.5 | 370 ± 70 |
| 2 | Fish 2 | 37.5 ± 0.2 | 590 ± 70 |
| 3 | Fish 3 | 38.5 ± 0.3 | 570 ± 90 |
| 4 | Fish 3 | 39.1 ± 0.1 | 770 ± 60 |
| 5 | Fish 4 | 37.1 ± 0.3 | 440 ± 50 |
| 6 | Fish 5 | 37.8 ± 0.1 | 570 ± 30 |
| 7 | Fish 6 | 37.0 ± 0.2 | 570 ± 50 |
| 8 | Fish 7 | 36.4 ± 0.3 | 550 ± 80 |
| 9 | Fish 8 | 37.3 ± 0.2 | 550 ± 80 |
| 10 | Fish 8 | 38.3 ± 0.2 | 580 ± 50 |
| | **MEAN** | **37.6 ± 0.8** | **600 ± 100** |

**Supplementary Table 2**. Thermodynamic parameters and standard deviations derived from a two-state fit of the equilibrium eye lens data. *Were identified as outliers as described above.

| Eye Lens Cell # | Organism # | $T_m$ (°C) | $\Delta H$ (kcal mol$^{-1}$) |
|---|---|---|---|
| 1 | Fish 1 | 40.5 ± 0.4 | 700 ± 200 |
| 2 | Fish 2 | 41.5 ± 0.2 | 420 ± 30 |
| 3 | Fish 3 | 41.0 ± 0.1 | 580 ± 30 |
| 4* | Fish 4 | 37.6 ± 0.3 | 800 ± 200 |
| 5 | Fish 5 | 40.2 ± 0.2 | 450 ± 40 |
| 6 | Fish 6 | 40.5 ± 0.2 | 500 ± 40 |
| 7 | Fish 7 | 40.6 ± 0.1 | 460 ± 10 |
| 8* | Fish 8 | 38.0 ± 0.4 | 700 ± 200 |
| 9 | Fish 9 | 40.2 ± 0.4 | 500 ± 100 |
| 10 | Fish 10 | 40.5 ± 0.3 | 460 ± 60 |
| 11 | Fish 10 | 39.9 ± 0.1 | 660 ±50 |
| | **MEAN** | **40.5 ± 0.5** | **500 ± 100** |

**Supplementary Table 3**. Thermodynamic parameters and standard deviations derived from a two-state fit of the equilibrium keratinocyte data. *Were identified as outliers as described above.

| Keratinocyte Cell # | Organism # | $T_m$ (°C) | $\Delta H$ (kcal mol$^{-1}$) |
|---|---|---|---|
| 1 | Fish 1 | 37.8 ± 0.2 | 570 ± 70 |
| 2 | Fish 1 | 37.8 ± 0.2 | 550 ± 60 |
| 3 | Fish 1 | 37.8 ± 0.2 | 640 ± 90 |
| 4 | Fish 1 | 37.7 ± 0.3 | 540 ± 60 |
| 5 | Fish 2 | 36.8 ± 0.3 | 410 ± 50 |
| 6 | Fish 3 | 39.6 ± 0.3 | 380 ± 30 |
| 7* | Fish 4 | 41.1 ± 0.1 | 350 ± 30 |
| 8* | Fish 4 | 41.1 ± 0.1 | 380 ± 10 |
| 9 | Fish 5 | 37.8 ± 0.1 | 640 ± 60 |
| 10 | Fish 5 | 38.4 ± 0.1 | 530 ± 30 |
| 11 | Fish 6 | 40.8 ± 0.4 | 340 ± 50 |
| 12 | Fish 7 | 37.2 ± 0.1 | 550 ± 60 |
| 13 | Fish 8 | 40.0 ± 0.2 | 420 ± 20 |
| 14 | Fish 9 | 37.9 ± 0.2 | 500 ± 50 |
| 15 | Fish 9 | 39.6 ± 0.3 | 380 ± 40 |
| 16 | Fish 9 | 38.0 ± 0.2 | 470 ± 40 |
| **MEAN** | | **38 ± 1** | **500 ± 100** |

**Supplementary Table 4.** Thermodynamic parameters and standard deviations derived from a two-state fit of the equilibrium notochord data.

| Notochord Cell # | Organism # | $T_m$ (°C) | $\Delta H$ (kcal mol$^{-1}$) |
|---|---|---|---|
| 1 | Fish 1 | 38.7 ± 0.1 | 790 ± 80 |
| 2 | Fish 2 | 39.19 ± 0.09 | 680 ± 40 |
| 3 | Fish 3 | 39.1 ± 0.1 | 760 ± 60 |
| 4 | Fish 4 | 39.6 ± 0.2 | 640 ± 60 |
| 5 | Fish 5 | 38.7 ± 0.1 | 530 ± 30 |
| 6 | Fish 6 | 37.2 ± 0.3 | 660 ± 90 |
| 7 | Fish 7 | 37.4 ± 0.3 | 570 ± 80 |
| 8 | Fish 8 | 38.6 ± 0.2 | 520 ± 50 |
| 9 | Fish 9 | 39.3 ± 0.1 | 540 ± 30 |
| 10 | Fish 10 | 38.7 ± 0.4 | 570 ± 90 |
| 11 | Fish 11 | 38.5 ± 0.2 | 590 ± 50 |
| | **MEAN** | **38.6 ± 0.7** | **600 ± 90** |

**Supplementary Table 5.** Thermodynamic parameters and standard deviations derived from a two-state fit of the equilibrium U-2 OS mammalian cell data. *Were identified as outliers as described above.

| U-2 OS Cell # | $T_m$ (°C) | $\Delta H$ (kcal mol$^{-1}$) |
|---|---|---|
| 1 | 34.0 ± 0.2 | 520 ± 60 |
| 2 | 34.8 ± 0.1 | 570 ± 10 |
| 3 | 35.0 ± 0.1 | 540 ± 50 |
| 4 | 35.3 ± 0.1 | 550 ± 30 |
| 5 | 35.5 ± 0.4 | 520 ± 90 |
| 6 | 33.2 ± 0.2 | 610 ± 80 |
| 7 | 33.4 ± 0.2 | 580 ± 90 |
| 8 | 34.8 ± 0.2 | 640 ± 70 |
| 9 | 33.5 ± 0.2 | 640 ± 80 |
| 10 | 33.5 ± 0.1 | 490 ± 20 |
| 11 | 33.5 ± 0.1 | 480 ± 30 |
| 12 | 34.7 ± 0.2 | 530 ± 60 |
| 13 | 34.3 ± 0.2 | 600 ± 60 |
| 14 | 35.1 ± 0.2 | 600 ± 70 |
| 15 | 35.6 ± 0.3 | 490 ± 70 |
| 16 | 36.3 ± 0.1 | 490 ± 30 |
| 17 | 35.2 ± 0.1 | 430 ± 30 |
| 18* | 40.0 ± 0.2 | 480 ± 30 |
| 19 | 35.9 ± 0.1 | 570 ± 20 |
| 20 | 38.4 ± 0.4 | 350 ± 50 |
| **MEAN** | **35 ± 1** | **540 ± 70** |

**Supplementary Table 6.** Thermodynamic parameters and standard deviations of Construct 1 (no loop mutation) derived from a two-state fit of the equilibrium myocyte data. Because there is no post-transition baseline $\Delta H$ was fixed to 60 kcal mol$^{-1}$. *Were identified as outliers as described above.

| Myocyte Cell # | Organism # | $T_m$ (°C) |
|---|---|---|
| 1 | Fish 1 | 41.3 ± 0.7 |
| 2 | Fish 2 | 41.4 ± 0.7 |
| 3 | Fish 3 | 42.4 ± 0.7 |
| 4 | Fish 4 | 41.9 ± 0.3 |
| 5 | Fish 5 | 41.3 ± 0.9 |
| 6 | Fish 6 | 41 ± 3 |
| 7 | Fish 7 | 42.6 ± 0.6 |
| 8* | Fish 8 | 45.7 ± 0.6 |
| | **MEAN** | **41.6 ± 0.6** |

**Supplementary Table 7.** Thermodynamic parameters and standard deviations of Construct 1 (no loop mutation) derived from a two-state fit of the equilibrium U-2 OS mammalian cell data.

| U-2 OS Cell # | $T_m$ (°C) | $\Delta H$ (kcal mol$^{-1}$) |
|---|---|---|
| 1 | 37.8 ± 0.4 | 400 ± 60 |
| 2 | 38.2 ± 0.4 | 350 ± 50 |
| 3 | 37.0 ± 0.4 | 410 ± 70 |
| 4 | 37.5 ± 0.2 | 380 ± 40 |
| 5 | 36.8 ± 0.5 | 420 ± 70 |
| 6 | 42.4 ± 0.7 | 320 ± 30 |
| 7 | 40.48 ± 0.06 | 384 ± 7 |
| 8 | 37.9 ± 0.3 | 380 ± 50 |
| 9 | 40.5 ± 0.5 | 390 ± 70 |
| 10 | 37.0 ± 0.3 | 380 ± 40 |
| 11 | 38.3 ± 0.2 | 420 ± 50 |
| 12 | 40.6 ± 0.2 | 380 ± 20 |
| 13 | 39.6 ± 0.2 | 440 ± 30 |
| 14 | 35.8 ± 0.2 | 370 ± 30 |
| 15 | 38.9 ± 0.2 | 370 ± 20 |
| **MEAN** | **39 ± 2** | **400 ± 30** |

**Supplementary Table 8.** Myocyte relaxation lifetimes and standard deviations derived from a single exponential fit (Equation 6) of observed transients following a jump to $T_m$. One cell was analyzed per fish.

| Myocyte Fish # | $\tau$ (s) |
|---|---|
| 1 | 1.6 ± 0.1 |
| 2 | 1.59 ± 0.06 |
| 3 | 1.96 ± 0.07 |
| 4 | 1.58 ± 0.06 |
| 5 | 0.93 ± 0.05 |
| 6 | 1.2 ± 0.1 |
| 7 | 1.17 ± 0.06 |
| 8 | 1.64 ± 0.05 |
| 9 | 1.14 ± 0.05 |
| 10 | 0.88 ± 0.07 |
| 11 | 1.48 ± 0.02 |
| 12 | 1.17 ± 0.04 |
| **MEAN** | **1.4 ± 0.3** |

**Supplementary Table 9.** Keratinocyte relaxation lifetimes and standard deviations derived from a single exponential fit (Equation 6) of observed transients following a jump to $T_m$. One cell was analyzed per fish.

| Keratinocyte Fish # | $\tau$ (s) |
|---|---|
| 1 | 0.9 ± 0.2 |
| 2 | 1.2 ± 0.6 |
| 3 | 1.16 ± 0.08 |
| 4 | 1.6 ± 0.9 |
| 5 | 1.0 ± 0.1 |
| 6 | 1.5 ± 0.2 |
| 7 | 1.4 ± 0.3 |
| 8 | 1.8 ± 0.2 |
| 9 | 0.7 ± 0.2 |
| 10 | 1.3 ± 0.8 |
| **MEAN** | **1.3 ± 0.3** |

**Supplementary Table 10.** Eye lens cell relaxation lifetimes and standard deviations derived from a single exponential fit (Equation 6) of observed transients following a jump to $T_m$. One cell was analyzed per fish.

| Eye Lens Fish # | $\tau$ (s) |
|---|---|
| 1 | 1.9 ± 0.3 |
| 2 | 1.8 ± 0.3 |
| 3 | 1.7 ± 0.3 |
| 4 | 1.7 ± 0.3 |
| 5 | 1.81 ± 0.06 |
| 6 | 1.59 ± 0.04 |
| 7 | 2.1 ± 0.1 |
| 8 | 1.6 ± 0.4 |
| 9 | 1.76 ± 0.05 |
| 10 | 2.1 ± 0.1 |
| **MEAN** | **1.8 ± 0.2** |

**Supplementary Table 11.** U-2 OS relaxation lifetimes and standard deviations derived from a single exponential fit (Equation 6) of observed transients following a jump to $T_m$.

| U-2 OS Cell # | $\tau$ (s) |
|---|---|
| 1 | 1.6 ± 0.5 |
| 2 | 1.6 ± 0.5 |
| 3 | 1.5 ± 0.5 |
| 4 | 1.6 ± 0.2 |
| 5 | 1.4 ± 0.5 |
| 6 | 0.94 ± 0.07 |
| 7 | 0.9 ± 0.2 |
| 8 | 1.1 ± 0.4 |
| 9 | 1.2 ± 0.1 |
| 10 | 1.8 ± 0.4 |
| 11 | 1.53 ± 0.4 |
| 12 | 1.3 ± 0.1 |
| 13 | 1.42 ± 0.08 |
| **MEAN** | **1.4 ± 0.3** |

## Supplementary Methods

### Temperature jumps

In principle the temperature jump approach can be applied to measure protein dynamics in any transparent living organism. Objectives must be selected with a long free working distance for thicker specimen and with an appropriate magnification for the cell type of interest. Our temperature-jump approach requires objectives that are not immersed in fluid, because heat will dissipate more quickly through the immersion fluid (oil, water, glycerin) than the air.

The thickness of the sample can impact (1) the duration of the T-jump and (2) the uniformity of heating through the sample:

(1) The duration of the T-jump is determined by thermal diffusion out of the heated volume. The advantage of the programmable laser is that the temperature profile can be adjusted so that the temperature following the jump is constant within ±0.25 °C. The power density at the sample was controlled by a TTL voltage input provided by LabView.

(2) The sample heating can change in the **x**, **y**, or **z** direction. Aligning the pump laser 90° normal to the sample ensures that there is no **x** or **y** drift in the heated volume with changes in **z**. The sample heating is not uniform with **z** because of the thickness of the sample.[1] This means that cells found at different heights in the zebrafish would experience a different size temperature jump. To overcome this, we designed three laser output power profiles at 0, 135 or 270 μm above the coverslip to achieve a consistent ~4 °C temperature-jump.

### Sensitivity to transient interactions

To test the sensitivity of PGK to transient sticking interactions we designed a surface modulated variant of FRET-PGK (Construct 1) where the loop insertion was mutated back to wildtype. The flexibility of the N-terminal where the loop is located is unaffected by

loop insertion[2], so removal of the loop will not impact the sensitivity of PGK to macromolecular crowding. On the other hand, the loop is located at the surface of PGK and contains a polar serine group, so removal of the loop could impact the way PGK interacts transiently with the environment.

We compared the stability of FRET-PGK with and without the loop in two different cell types, mammalian U-2 OS cells and zebrafish myocytes (Supplementary Table 1, 5-7). If transient interactions arising from the different local environments contribute to in-cell stability, we would expect the difference between the stability of the two proteins in U-2 OS cells to be different from myocytes. Instead, we observe that the onset of unfolding of the two constructs differs by ≈4 °C in both cell types (Supplementary Table 1, 5-7, Supplementary Figure 4). This demonstrates that PGK is relatively insensitive to transient interactions.

**Supplementary References**


1. Kubelka, J. Time-resolved methods in biophysics. 9. Laser temperature-jump methods for investigating biomolecular dynamics. *Photochem. Photobiol. Sci.* **8,** 499–512 (2009).

2. Collinet, B., Garcia, P., Minard, P. & Desmadril, M. Role of loops in the folding and stability of yeast phosphoglycerate kinase. *Eur. J. Biochem.* **268,** 5107–5118 (2001).
