# Supplementary material for: Quantifying protein dynamics and stability in a living organism
Source: Nat Commun. 2019 Mar 12;10:1179. doi: 10.1038/s41467-019-09088-y (PMC6414637; doi:10.1038/s41467-019-09088-y)
Supplement: Supplementary file 3 — Reporting Summary [file 41467_2019_9088_MOESM3_ESM.pdf]

nature research

Corresponding author(s): Martin Gruebele and Caitlin M. Davis

Reporting Summary

Nature Research wishes to improve the reproducibility of the work that we publish. This form provides structure for consistency and transparency in reporting. For further information on Nature Research policies, see Authors & Referees and the Editorial Policy Checklist.

Statistical parameters

When statistical analyses are reported, confirm that the following items are present in the relevant location (e.g. figure legend, table legend, main text, or Methods section).

n/a	Confirmed	
☐	☒	The exact sample size (n) for each experimental group/condition, given as a discrete number and unit of measurement
☐	☒	An indication of whether measurements were taken from distinct samples or whether the same sample was measured repeatedly
☐	☒	The statistical test(s) used AND whether they are one- or two-sided<br>*Only common tests should be described solely by name; describe more complex techniques in the Methods section.*
☒	☐	A description of all covariates tested
☐	☒	A description of any assumptions or corrections, such as tests of normality and adjustment for multiple comparisons
☐	☒	A full description of the statistics including central tendency (e.g. means) or other basic estimates (e.g. regression coefficient) AND variation (e.g. standard deviation) or associated estimates of uncertainty (e.g. confidence intervals)
☒	☐	For null hypothesis testing, the test statistic (e.g. F, t, r) with confidence intervals, effect sizes, degrees of freedom and P value noted<br>*Give P values as exact values whenever suitable.*
☒	☐	For Bayesian analysis, information on the choice of priors and Markov chain Monte Carlo settings
☒	☐	For hierarchical and complex designs, identification of the appropriate level for tests and full reporting of outcomes
☒	☐	Estimates of effect sizes (e.g. Cohen's d, Pearson's r), indicating how they were calculated
☒	☐	Clearly defined error bars<br>*State explicitly what error bars represent (e.g. SD, SE, CI)*

Our web collection on statistics for biologists may be useful.

Software and code

Policy information about availability of computer code

Data collection	The microscope was controlled using LabView 13.0.1f2 (National Instruments).
Data analysis	Image analysis was performed in MATLAB R2016b (MathWorks). Thermodynamic and kinetic fitting were performed in Igor Pro version 8.02 (WaveMetrics).

For manuscripts utilizing custom algorithms or software that are central to the research but not yet described in published literature, software must be made available to editors/reviewers upon request. We strongly encourage code deposition in a community repository (e.g. GitHub). See the Nature Research guidelines for submitting code & software for further information.

Data

Policy information about availability of data

All manuscripts must include a data availability statement. This statement should provide the following information, where applicable:

- Accession codes, unique identifiers, or web links for publicly available datasets
- A list of figures that have associated raw data
- A description of any restrictions on data availability

Complete tables of the equilibrium thermodynamic and kinetic parameters of myocyte, keratinocyte, eye lens, notochord, and U-2 OS cells are available in the

supporting information. The source data underlying Figs 2b, c, 3 and Supplementary Figs S1-S4 are provided as a source data file. Additional data that support the findings of this study are available from the corresponding author upon reasonable request.

Field-specific reporting

Please select the best fit for your research. If you are not sure, read the appropriate sections before making your selection.

☒ Life sciences ☐ Behavioural & social sciences ☐ Ecological, evolutionary & environmental sciences

For a reference copy of the document with all sections, see nature.com/authors/policies/ReportingSummary-flat.pdf

Life sciences study design

All studies must disclose on these points even when the disclosure is negative.

Sample size	We collected at least 10 samples for each cell type (myocyte, eye lens, keratinocyte, and notochord) and for each assay (stability and kinetics). This number allows a reasonably accurate estimate of cell-to-cell variance of cooperativity, melting temperature and kinetics within a given tissue.
Data exclusions	Prior to fluorescence imaging the animals with any visible developmental abnormality were removed. Because the FRET-labeled proteins were expressed in a mosaic fashion in each fish and across different individuals, we inspected the animals under a fluorescence microscope and selected the fish that had at least one cell with sufficient fluorescence for experiments. During the heating sessions, a small fraction of the fish did not survive the high temperature and were excluded from the data analysis. These exclusion criteria were established prior to any data collection for the study.
Replication	For each experimental group, the data presented in this study were collected on multiple animals and during an extended period of time spanning several weeks.
Randomization	The animals were allocated into different experimental groups based on time. We repeated the same gene transformation and fluorescence measurements on one cell type until the sample size for this cell type meets our pre-determined criterion, which is 10 for each cell type. The experiments for each cell type took several weeks. This allocation was not random, but the covariate (time in the year) is not relevant to the study because the animals were kept under the same lab conditions (constant temperature, same light/dark cycle) throughout the year.
Blinding	Blinding was not possible in this study. Human involvement was necessary during sample selection.

Reporting for specific materials, systems and methods

Materials & experimental systems

n/a	Involved in the study	
☒	☐	Unique biological materials
☒	☐	Antibodies
☐	☒	Eukaryotic cell lines
☒	☐	Palaeontology
☐	☒	Animals and other organisms
☒	☐	Human research participants

Methods

n/a	Involved in the study	
☒	☐	ChIP-seq
☒	☐	Flow cytometry
☒	☐	MRI-based neuroimaging

Eukaryotic cell lines

Policy information about cell lines

Cell line source(s)	U-2 OS (ATCC HTB-96)
Authentication	ATCC provided a certificate of analysis of authentication.
Mycoplasma contamination	ATCC tested for mycoplasma contamination and none was detected.
Commonly misidentified lines (See ICLAC register)	No commonly misidentified lines were used.

Animals and other organisms

Policy information about studies involving animals; ARRIVE guidelines recommended for reporting animal research

Laboratory animals	Danio rerio, AB, male and female, embryos and 2 days post fertilization
Wild animals	The study did not involve wild animals.
Field-collected samples	The study did not involve samples collected from the field.
